# Experience with and perceptions of non-prescription anthelmintics for cancer treatments among cancer patients in South Korea: A cross-sectional survey

**DOI:** 10.1371/journal.pone.0275620

**Published:** 2022-10-04

**Authors:** Bomi Song, Kwang Joon Kim, Sung Hwan Ki

**Affiliations:** 1 Graduate School of Clinical Pharmacy, Chosun University, Gwangju, Republic of Korea; 2 College of Pharmacy, Mokpo National University, Jeonnam, Republic of Korea; 3 College of Pharmacy, Chosun University, Gwangju, Republic of Korea; UT MD Anderson Cancer Center, UNITED STATES

## Abstract

Although non-prescription anthelmintics are used by many patients as cancer treatment in South Korea, data regarding the experiences or perceptions of these drugs are lacking. This study aimed to investigate the repercussions of non-prescription anthelmintics for cancer treatment and evaluate their perceived effectiveness and adverse effects. This survey included 86 cancer patients, aged 19 years and older, who underwent anthelmintic therapy for cancer. They were recruited from two online communities in South Korea through a structured questionnaire that was provided online. Cancer patients under non-prescription anthelmintic therapy for cancer in South Korea were mostly in their advanced stages and had started the treatment in 2019. About half of the cancer patients had taken non-prescription anthelmintics during their chemotherapy, and 96.5% of them did not inform the clinicians. These participants had a positive perception (79.1%) toward the effectiveness of anthelmintics, as they felt it improved their physical condition. Data on the adverse effects of anthelmintics showed that more than two-third of the participants did not report experiencing any adverse effects. Communication between the clinicians and cancer patients regarding the use of non-prescription anthelmintics should be enhanced to prevent adverse effects.

## Introduction

The term “drug repurposing” or “drug repositioning” refers to the novel use of a drug previously developed or approved for a specific clinical purpose to treat a disease for which it was not originally designed [1]. In general, drug repurposing occurs when a particular disease has few remedies coupled with exceedingly high demand. This phenomenon is common in the field of medicine and can be attributed to the long period of time required for, and the high cost of, drug development [2, 3]. If existing approved drugs can be made available within a shorter time period by decreasing the number of required clinical trials and reducing the number of process validations and stability tests, patients can rapidly access an additional treatment option at a reasonable price.

Currently, drug repurposing is being studied as a novel strategy in various areas of drug development [1] including COVID-19, cardiovascular diseases, pulmonary arterial hypertension, and cancer. In recent years, drug repurposing for COVID-19 therapies has increased rapidly, owing to the publishing of more than hundreds of studies every year. Four major groups of drugs are being developed through drug repurposing for COVID-19 treatment: antivirals (lopinavir/ritonavir, oseltamivir, and remdesivir), immunosuppressors (eculizumab, dexamethasone, and budesonide), immunomodulators (camostat, interferons, and sargramostim), and other well-known drugs (azithromycin, doxycycline, and nitazoxanide) [4]. One example of drug repurposing is the use of antidiabetic drugs, such as sodium-glucose cotransporter 2 inhibitor (dapagliflozin) and glucagon-like peptide-1 receptor agonists (liraglutide and semaglutide) as potential cardiovascular drugs [5]. These medications were found to reduce the risk of cardiovascular disorders in people with or without diabetes. Another successful example of repurposing includes the use of bosentan, iloprost, and sildenafil for the treatment of pulmonary arterial hypertension, which often causes serious outcomes [6].

Since there are no non-toxic and effective standardized medications for cancer, numerous studies on drug repurposing for cancer treatment are progressing as well. Zhang et al. grouped the cancer therapeutic repurposing drugs into 10 groups based on their potential to inhibit the following cancer hallmarks: sustaining proliferative signaling (e.g., rapamycin and prazosin), evading growth suppressors (ritonavir, etc.), withstanding cell death (artemisinin, etc.), inducing replicative immortality (curcumin, etc.), genome instability and mutation (mebendazole, etc.), reprogramming energy metabolism (metformin, etc.), inducing angiogenesis (itraconazole, etc.), activating invasion and metastasis (niclosamide, etc.), tumor-promoting inflammation (aspirin, etc.), and evading immune destruction (infectious disease vaccines) [3].

Recently in South Korea, a controversy about the anticancer potential of non-prescription anthelmintics emerged when a man named Joe Tippens claimed to have completely cured his lung cancer by taking a dog-deworming drug (communicated through YouTube in 2019), with fenbendazole as its active pharmaceutical ingredient [7]. Joe Tippens used the following treatment regimen: curcumin, 600 mg per day; cannabidiol oil, 25 mg per day; and fenbendazole, 222 mg per day for 3 consecutive days with four-day intervals in between [8]. Following the appearance of the YouTube video, pharmacies experienced shortages of anthelmintics, including fenbendazole, for several months due to the sudden increase in demand for these drugs. The use of anthelmintics in cancer patients without the consent of, or prescriptions from, medical institutions continued. Although many cancer patients continue to take non-prescription anthelmintics, no study has reported on the perceptions and actual experiences of patients using this medication.

Thus, this study aimed to understand the medication methods, perceptions of anticancer efficacy, and adverse effects of non-prescription anthelmintics among cancer patients in South Korea. To this end, a structured survey was conducted to collect data from cancer patients who had taken non-prescription anthelmintics to treat their cancer.

## Methods

### Samples and settings

Cancer patients were recruited from two online mega communities in South Korea; the largest ones have more than 2,000 members and were established to exchange information about the use of anthelmintics. The recruitment notice was advertised on the community webpages, and volunteers willing to participate in the survey contacted the representative research team. A survey link was sent to the volunteers through online chat boxes to prevent unauthorized members from accessing the link. The sample included cancer patients aged 19 years and older who had taken anthelmintics as cancer treatment. Among the 168 participants, only 86 completed the survey. The survey was a structured questionnaire containing 28 questions (6 on general characteristics, 21 on the survey topics, and 1 for free expression of the participants’ opinions) on the online platform DOOIT Survey (DOOIT, Seoul, South Korea) and was conducted from April 2021 to July 2021. It took approximately 30 min for each participant to complete the survey. The detailed survey questionnaire can be found in S1 File.

### Survey structure

This survey was largely divided into six parts: i) the characteristics of patients, ii) the methods of anthelmintics administration, iii) effectiveness of anthelmintics, iv) adverse effects of anthelmintics, v) communications with clinicians, and vi) free description on their experiences. In the first part, data on the patients’ characteristics, including their gender, age, education, and cancer diagnosis were collected. In the second part, the duration and mode of anthelmintics administration, the distributor of the drugs, name of the drugs, and dosage of the drugs were investigated. In the third part, the patients’ perceptions regarding the antitumor efficacy of anthelmintics and the reasons for their evaluation were determined. The fourth part explored the adverse effects and details on the type, frequency, duration, and severity of the adverse effects. In the fifth part, information regarding clinician’s support, comments, and consent regarding the usage of anthelmintics were evaluated. Finally, in the sixth part, all participants were provided a chance to share their experiences of taking anthelmintics.

### Statistics

The survey responses were downloaded from DOOIT Survey in a Microsoft Excel 2010 (Microsoft Co., Redmond, WA, USA) file format. The analysis was performed in the IBM SPSS Statistics 25 program (IBM Co., Armonk, NY, USA). Categorical variables were expressed as frequencies and percentages, while continuous variables, including age or duration, were expressed as mean, standard deviation, and median.

### Ethics approval

Approval was obtained on April 20, 2021 from the Institutional Review Board at Chosun University, South Korea (IRB) (IRB no.: 2-1041055-AB-N-01-2021-7). The procedures in this study were performed in accordance with the tenets of the Declaration of Helsinki.

### Informed consent

The requirement for written informed consent was waived by the IRB. Obtaining consent was practically impossible during the research process due to the fact that the participants were members of online communities. Thus, in order to confirm informed consent, a written agreement was obtained from all individual participants with the instructions presented prior to the survey by asking them to select “agree” or “not agree.”

## Results

### Patients’ general characteristics

Of the total 168 participants, only 86 completed the survey; the general characteristics of the patients are described in Table 1. The patients included 40 men and 46 women, with a mean age of 55.2±13.0 years. Among the participants, 56 (65.1%) were college graduates. The participants had been diagnosed with the following different types of cancer: breast (20.9%), lung (10.5%), intestinal (10.5%), liver (8.1%), and gastric (5.8%). Most patients (74.4%) had been diagnosed with cancer between 2016 and 2020, and 52.3% of patients were diagnosed as having stage 4 cancer.

**Table 1:** Patients’ general characteristics.

Characteristics		Total sample	%
		N = 86	
Age (years)	Average±*SD*	55.2±13.0	
	Min-max (median)	19–90 (55)	
	19	1	1.2
	20–29	0	0
	30–39	8	9.3
	40–49	22	25.6
	50–59	20	23.3
	60–69	22	25.6
	70–79	11	12.8
	80–89	1	1.2
	90–99	1	1.2
Gender	Male	40	46.5
	Female	46	53.5
Education status	No education	2	2.3
	Elementary school graduate	3	3.5
	Middle school graduate	4	4.7
	High school graduate	21	24.4
	College graduate	56	65.1
Diagnosis	Lung	9	10.5
	Gastric	5	5.8
	Liver	7	8.1
	Intestinal	9	10.5
	Breast	18	20.9
	Others	38	44.2
Stage	1	9	10.5
	2	14	16.3
	3	18	20.9
	4	45	52.3
Time of diagnosis (year)	1996–2000	2	2.3
	2001–2005	1	1.2
	2006–2010	3	3.5
	2011–2015	13	15.1
	2016–2020	64	74.4
	2021–	3	3.5

### Taking anthelmintics as cancer treatment

Details regarding the use of anthelmintics as a cancer treatment are presented in Table 2. Approximately 96.5% of the patients started taking anthelmintics from 2019, the year when Joe Tippens’ video interview was released to the public. The mean duration from the time of cancer diagnosis until the onset of treatment with anthelmintics was 27.5 months. The mean duration of anthelmintic usage was 10.5±7.8 months. Almost half (48.8%) of the participants indicated that they had taken anthelmintics while they were undergoing chemotherapy. Most participants (64.0%) reported that they continued to use anthelmintic drugs at the time when they participated in the survey.

**Table 2:** Details of anthelmintic use for cancer treatment.

Survey question	Response	Total sample	%
		N = 86	
1. When did you begin taking anthelmintics for cancer treatment? (year)	1996–2000	1	1.2
	2001–2005	1	1.2
	2006–2010	0	0
	2011–2015	0	0
	2016–2020	78	90.7
	2021–	6	7.0
2. Please mention the approximate duration (months) of treatment with anthelmintics.	Average±*SD*	10.5±7.8	
	Min-Max (median)	1–44 (10)	
3. Please select the period(s) during which you have taken anthelmintics.	After diagnosis, before chemotherapy	25	29.1
	During chemotherapy	42	48.8
	Resting chemotherapy	20	23.3
	Discontinuing chemotherapy	11	12.8
4. Are you still taking anthelmintics?	Yes	55	64.0
	No	31	36.0
5. Please mention what motivated you to take anthelmintics.	Information from TV news	5	5.8
	Information from YouTube	36	41.9
	Information from online news	10	11.6
	Information from online communities	22	25.6
	Information from acquaintances	5	5.8
	Recommendation of a clinician	3	3.5
	Others	5	5.8
6. From where did you purchase anthelmintics?	Local pharmacy	39	45.3
	Purchased online	65	75.6
	Others	14	16.3
7. Please mention the name of the anthelmintic you used for cancer treatment.	Albendazole	52	60.5
	Fenbendazole	45	52.3
	Flubendazole	5	5.8
	Mebendazole	42	48.8
	Triclabendazole	1	1.2
	Niclosamide	30	34.9
	Nitazoxanide	14	16.3
	Praziquantel	5	5.8
	Ivermectin	54	62.8
	Pyrvinium	8	9.3
	Do not know	1	1.2
8. Please choose the medication method of anthelmintics.	Daily without resting	31	36.0
	In a routine schedule with resting	54	62.8
	Intermittently	7	8.1
	Others	9	10.5
9. Please choose the daily dosing regimen of the anthelmintics.	Once	26	30.2
	Twice	50	58.1
	More than three times	21	24.4
	Do not know	0	0

The participants responded that they selected anthelmintics as a therapeutic option for the following reasons: information available on social media, such as YouTube (41.9%); online communities (25.6%); or online news (11.6%). Moreover, they preferred shopping online (75.6%) for anthelmintics rather than purchasing them from community pharmacies (45.3%). The most preferred anthelmintic of the survey participants was ivermectin (62.8%), which belongs to the non-benzimidazole group, followed by albendazole (60.5%) and fenbendazole (52.3%). The patients used an average of three types of anthelmintics, each with different active pharmaceutical ingredients, either alone or in combination. The maximum number of anthelmintics taken by a patient was eight. More than half of the patients (62.8%) reported that they took the medicines on a schedule; that is, they took medicine on consecutive days followed by a drug holiday of several days. The most common frequency of medicine intake was twice a day (58.1%).

### Perceptions on the anticancer efficacy of anthelmintics

Perceptions on the anticancer efficacy of anthelmintics are described in Table 3. Most patients (79.1%) answered “yes” when they were asked whether they perceived anthelmintic therapy to be effective in their cancer treatment. When the participants were asked why they thought the treatment was effective, 52.3% indicated that the treatments improved their perceived physical conditions. Moreover, 34.9% and 3.5% of the participants stated they perceived that the anthelmintic therapy reduced the spread of cancer in the affected area and decreased the number of cancerous masses, respectively. In contrast, 20.9% of participants chose “no” in response to the same question because they thought that anthelmintics use worsened their cancer status (9.3%) or had no effect (7.0%).

**Table 3:** Perceptions on the antitumor efficacy of anthelmintics.

Survey question	Response	Total sample	%
		N = 86	
10. Do you think the anthelmintics were effective in treating cancer?	Yes	68	79.1
	No	18	20.9
11. If the anthelmintics taken were effective in cancer treatment, why do you think so?	Decline in tumor size	30	34.9
	Decrease in the number of tumor masses	3	3.5
	Improvement in physical condition	45	52.3
	Others	27	31.4
12. If the anthelmintics taken were ineffective in cancer treatment, why do you think so?	No change in cancer status	6	7.0
	Worsening cancer status	8	9.3
	Others	4	4.7

### Perceptions on the adverse effects of anthelmintics

Perceptions on the adverse effects of anthelmintics are shown in Table 4. The majority of participants (73.3%) responded “no” when asked whether they had experienced any adverse effects after taking anthelmintics as cancer treatment. For the participants who responded “yes” (26.7%), additional questions were asked to determine the nature of their side effects. In this regard, 7.0% of patients reported gastrointestinal side effects, 3.5% reported liver abnormalities, and 3.5% reported hematological effects. In terms of the frequencies of occurrence of adverse effects, 12.8% of the participants answered “more than three times.” When asked about the onset of adverse effects, 11.6% of participants answered “after a month,” and 7.0% answered “within a month.” When surveyed about the severity of the adverse effects, 10.5% of the participants answered “a bit uncomfortable,” while 9.3% answered “uncomfortable but endurable.” When asked about measures taken to relieve the adverse effects, 12.8% of the participants answered “discontinued anthelmintic course,” and 9.3% answered “continued to take the same anthelmintic despite the adverse effects.”

**Table 4:** Perceptions on adverse effects of anthelmintics.

Survey question	Response	Total sample	%
		N = 86	
13. Have you experienced any adverse effects after taking anthelmintics?	Yes	23	26.7
	No	63	73.3
14. Please choose an adverse effect you think was caused by anthelmintics use.	Gastrointestinal symptoms	6	7.0
	Liver abnormalities	3	3.5
	Hematological symptoms	3	3.5
	Others	16	18.6
15. Please choose the number of times that you have experienced adverse effects after taking anthelmintics.	Once	9	10.5
	Twice	3	3.5
	More than three times	11	12.8
16. Please mention when the adverse effects occurred since the first use of anthelmintics?	Within a day	3	3.5
	Within a week	5	5.8
	Within a month	6	7.0
	After a month	10	11.6
17. Please choose the duration of the adverse effects that you experienced.	A day	8	9.3
	A week	10	11.6
	A month	4	4.7
	More than a month	2	2.3
18. Please choose the severity of adverse effects you experienced.	Not uncomfortable, but worsening of hematological parameters	7	8.1
	A bit uncomfortable	9	10.5
	Uncomfortable, but endurable	8	9.3
	Very severe, interfering with daily life	2	2.3
19. Please select the action you took when you developed adverse effects.	Discontinued anthelmintics	11	12.8
	Changed the type of anthelmintics	4	4.7
	Continued the same anthelmintics	8	9.3
	Used different additional medicines	1	1.2
	Others	5	5.8

### Communication with clinicians

Details about communications between patients and clinicians are listed in Table 5. When asked whether their respective clinicians were aware of the anthelmintic use for cancer treatment, most patients (96.5%) answered “no.” For the three patients who replied “yes,” a follow-up question was asked about clinician support. Two (2.3%) stated that the clinicians did not provide any help, and the remaining one (1.2%) replied that the clinician recommended they avoid taking anthelmintics due to the possibility of liver toxicity.

**Table 5:** Details about the communications between patients and clinicians.

Survey question	Response	Total sample	%
		N = 86	
20. Have you informed your clinician about taking anthelmintics to treat your cancer?	Yes	3	3.5
	No	83	96.5
21. If you received any support from your clinician regarding the use of anthelmintics, please choose below.	Advice on choosing the anthelmintic type	0	0
	Guidance for anthelmintic medication	0	0
	Actions to deal with adverse effects of anthelmintics	0	0
	No support	2	2.3
	Others	1	1.2

### Descriptions of their experiences

Finally, when patients were required to describe their experiences with anthelmintic therapy as cancer treatment, most provided positive feedback. Some patients suggested that anthelmintics should be made available in combination with clinically approved chemotherapy medications in the future, after clinical trials or drug development, while a few others expressed concerns about the underlying adverse effects of anthelmintic therapy.

## Discussion

Although the use of non-prescription anthelmintic drugs is prevalent in patients with cancer, there is a lack of information regarding patient behaviors or perceptions associated with these drugs. This study was the first to investigate factors leading to the use of non-prescription anthelmintics as treatment for cancer, such as motivation, mode of intake, and types of anthelmintics, and patient perceptions on their effectiveness in cancer treatment and adverse effects in cancer patients in South Korea. The survey participants were mainly college graduates (65.1%), and most (73.3%) had advanced-stage cancers. The results showed that a large proportion of participants were dependent on information from social media or online platforms when they decided to start anthelmintic therapy or attempted to purchase these medicines. Most patients (96.5%) revealed that they started taking anthelmintics in 2019, the year when Joe Tippens released his video; overall, 42% of patients reported that the information on YouTube motivated them to try anthelmintics. Based on these two results, it can be speculated that the beginning of anthelmintic use in South Korea was mainly triggered by the YouTube video mentioned in the Introduction.

Even though the video strongly affected patients’ decisions to use anthelmintics, the survey results revealed that almost all cancer patients taking anthelmintics modified their treatment methods based on the opinions of others and through dissemination of available information. First, although Joe Tippens mentioned fenbendazole as an effective drug, other types of anthelmintics, including ivermectin (of the non-benzimidazole group) as well as albendazole and flubendazole (of the benzimidazole group), were also used. Second, almost all patients were knowledgeable about the generic names of these medicines, which suggested that they had made efforts to expand their knowledge about anthelmintic therapies. Third, 62.8% of the patients followed Joe Tippens’ weekly medication method, consisting of several consecutive days of medicine intake with a break of a few days each week. However, the frequency of daily medicine intake was different from that of Joe Tippens’ regimen. A total of 58.1% of the patients took anthelmintics twice a day, while Joe Tippens took 1 g of canine granules (222 mg fenbendazole) per day. His regimen did not include a detailed description about the recommended daily frequency of drug intake. These results imply that the patients modified the anthelmintic regimen for cancer treatment and did not adhere to Tippens’ method mentioned in the Introduction.

In terms of perceptions about anthelmintic efficacy, more than two-thirds of the patients considered anthelmintic therapy to be an effective method of cancer treatment. Positive perceptions on its efficacy were related to the perceived improvements in their physical condition (52.3%), reductions in tumor sizes (34.9%), or decreases in the number of tumor masses (3.5%).

The most frequent type of adverse effect observed was gastrointestinal symptoms (7.0%); of the total cases, only 2.3% of patients indicated that these adverse effects were severe and affected their daily lives. More than two-thirds (73.3%) of the participants declared that they did not experience any adverse effects. This agrees with explanations in previous studies that anthelmintics, including benzimidazole derivatives, ivermectin, and praziquantel, are generally safe as demonstrated by the prolonged use of these medications [9–11]. However, in this study, several findings aroused concern regarding the adverse effects. First, since there was no medical guidance regarding the use of anthelmintic therapies for cancer treatment, the patients might have used higher-than-optimal doses. Considering that some severe cases were caused by the prolonged use of high doses of albendazole or praziquantel in patients with poor liver function [9], arbitrary decisions regarding dosages might lead some patients to develop adverse effects. Second, participants tended to use anthelmintics treatment on a long-term basis; the average duration of anthelmintics administration in all patients was 10.5 months. This raised concerns about the possibility of developing adverse effects from long-term intake. Third, the most patients (48.8%) responded that they took anthelmintics during their courses of chemotherapy. This result suggests that drug–drug interactions may be induced when anthelmintics are used in combination with chemotherapy. Fourth, the combination of anthelmintic therapies used by the participants might have also caused drug–drug interactions. For instance, one case report described a patient who developed psychosis after combined use of albendazole and ivermectin [12]. As albendazole and ivermectin were the anthelmintic medications preferred by the patients, this combination might have induced albendazole–ivermectin interactions. Fifth, despite the occurrence of adverse effects, some patients (9.3%) continued to take the same anthelmintics. These results suggest the necessity of clinician guidance regarding the safety of anthelmintic use to prevent harmful effects.

Most patients (96.5%) failed to inform their clinicians about the use of anthelmintics for cancer treatment. This result was consistent with that of previous studies [13, 14], which showed that a substantial proportion of patients refused to consult their clinicians about alternative therapies. Moreover, patients (3.5%) who informed their clinicians about the therapy reported that they did not receive their support. This information reflects the insufficiency of communication between cancer patients and their clinicians regarding the use of anthelmintics; thus, there is a need to highlight and spread awareness among the population regarding the safety of using anthelmintics for cancer treatment and promote seeking guidance from trained clinicians instead of an online community.

This study has several limitations that need to be addressed. First, since the recruitment and organization of the survey were performed online, the findings of this study may not be applicable to the general population of cancer patients, which includes those who are not familiar with internet platforms. Second, since many patients took anthelmintics while receiving chemotherapy, the combined effect of chemotherapy and anthelmintics could affect the results. Third, information about the exact dosages taken by the patients remains unknown because the questionnaire used multiple-choice questions. Finally, the survey only investigated patients’ perceptions, and there was not enough evidence or medical data available to evaluate the results.

## Conclusions

Communication between clinicians and cancer patients regarding the use of anthelmintics should be enhanced to prevent adverse effects. In addition, because anthelmintic medications can cause severe health issues, especially when used in high doses or combined with multiple regimens, cancer patients should be made aware of the risks of using non-prescribed anthelmintic medications. Furthermore, the government should be actively involved in investigating the blind spots in health security to spread awareness about off-label use of non-prescribed medications.

## Supporting information

S1 File Copy of the survey questionnaire. It contains data on the “recruitment of study participants” and detailed “survey questionnaire”.
(DOCX)
