# Supplementary material for: Experience with and perceptions of non-prescription anthelmintics for cancer treatments among cancer patients in South Korea: A cross-sectional survey
Source: PLoS One. 2022 Oct 4;17(10):e0275620. doi: 10.1371/journal.pone.0275620 (PMC9531786; doi:10.1371/journal.pone.0275620)
Supplement: S1 File — It contains data on the “recruitment of study participants” and detailed “survey questionnaire”. (DOCX) [file pone.0275620.s001.docx]

**연구참여자 모집**

**Recruitment of study participants**

본 연구에서는 항암목적으로 구충제를 복용한 경험이 있는 암환자를 대상으로 복용현황 및 부작용 발생 경험을 조사하는 연구로 조선대학교 임상약학대학원에서 석사학위 논문의 작성을 위해 진행하는 연구입니다. 위와 같은 사항에 대해 연구에 참여하실 분을 모집하고자 합니다.

This research investigates the status of medication methods and experience of adverse effects among cancer patients who have taken anthelmintics for cancer treatment. The data of this research will be used for a master's thesis at Graduate School of Clinical Pharmacy, Chosun University. We would like to recruit participants for this study.

**1. 연구제목 Study subject**

항암목적 구충제 사용 환자의 사용경험 및 이상반응 조사

Investigation of experience and adverse reactions of patients taking anthelmintics for cancer treatment

**2. 연구책임자 Study manager**

송보미 석사학위과정 (조선대학교 임상약학대학원)

Bomi Song/Master’s course (Graduate School of Clinical Pharmacy, Chosun University)

**3. 연구목적 Purpose of the study**

본 연구에서는 항암목적으로 구충제를 복용한 경험이 있는 암환자를 대상으로 복용현황 및 부작용 발생 경험을 조사하여 암치료 목적으로 사용되는 구충제의 안전성 관련 정보를 도출하고자 합니다.

This study aims to derive safety-related information on anthelmintics used for cancer treatment by examining the current status of medication methods and the experience of adverse effects in cancer patients who underwent anthelmintic therapy for cancer.

**4. 연구참여 선정기준 Criteria for selection of study participants**

만 19세 이상이며, 아래와 같은 시기에 항암목적으로 구충제를 복용한 경험이 있는 환자를 대상으로 합니다.

Patients aged 19 years and older who underwent anthelmintic therapy for cancer during the following periods will be recruited:

① 암을 진단받고 항암 약물치료를 시작하기 이전

② 암 치료를 위해 항암 약물치료와 병행하여

③ 암 치료를 마치고 항암 약물치료의 휴약(休藥) 상태일 때

④ 더 이상의 항암 약물치료가 불가하여 치료를 중단한 상태일 때

① After diagnosis, before chemotherapy

② During chemotherapy

③ Resting chemotherapy

④ Discontinuing chemotherapy

**5. 연구참여 제외기준 Criteria for exclusion of study participant**

① 설문에 참여 하였으나 설문에 대한 답변 내용이 명확하지 않은 지원자

② 연구책임자에게 참여 의사를 미리 밝히지 않은 카페 닉네임을 설문조사에 기입한 지원자

③ 설문조사에 기입한 네이버 ID가 실제 정보와 다른 지원자

① Applicants who participated in the survey, but whose answers are not directly relevant to the study

② Applicants who filled out the survey with a cancer community nickname without expressing the intention to participate to the study manager in advance

③ Applicants whose Naver ID entered in the survey is different from the actual ID

**6. 연구절차 Study procedure**

| **연구대상자 설명문 읽기**  **Reading instructions for study subjects** | ▶ | **설문조사 작성**  **Filling out the survey** | ▶ | **설문조사 제출**  **Submission of the survey** |
| --- | --- | --- | --- | --- |

**7. 참여절차 Participation procedure**

① 본 연구의 연구책임자인 송보미에게 카페 채팅을 통해 연구 참여 의사를 밝히면, 참여 의사를 밝히신 분에 한해 설문 링크를 안내해 드립니다.

② 안내 받은 설문 링크에 접속합니다.

③ 연구대상자 설명문을 읽은 후 설문조사 작성 및 제출합니다. (약 30분 이내 소요)

**① If a volunteer expresses his/her intention to participate in the study through community chat boxes to Bomi Song, the study manager, a survey link will be provided.**

**② Please access the survey link provided.**

**③ After reading instructions for study subjects, please fill out and submit the survey (It will take about 30 minutes).**

**8. 연구참여시 제공되는 사항 Rewards for participating in the study**

연구대상자 설명문을 읽고 설문조사를 작성하신 후 제출하신 모든 분(선착순 220명)에게 소정의 답례품(음료 기프티콘)을 드립니다.

A gift (beverage gift card) will be given to all those who have submitted the survey (for the 220 people on a first-come, first-served basis).

**9. 모집기간 및 참여방법 Recruitment period and participation method**

모집 기간은 IRB 승인일로부터 7월 31일까지 약 4개월 간 진행될 예정입니다. 본 연구의 연구대상자 수는 총 220명이며 연구대상자 모집이 선착순으로 마감되면 기간은 단축될 수 있습니다. 참여를 원하시는 분은 연구책임자 송보미에게 카페 채팅으로 연락해 주시기 바랍니다. 연구에 대한 자세한 설명을 보실 수 있고, 설명서를 읽은 후 설문조사가 시작됩니다.

The recruitment will be conducted for about 4 months from the date of approval of the Institutional Review Board to July 31, 2021. The total number of the subjects in this study is 220. If the study subjects are recruited earlier, the study period may be shortened. Interested participants can contact Bomi Song, the study manager, through community chat. The instructions will be sent via a link, and after reading the instructions, the participants can submit their survey responses.

**※ 기타문의**: 송보미 대학원생 (e-mail: *********@naver.com )

**※ Inquiry**: Bomi Song/ Graduate School Student (e-mail: *********@naver.com )

**설문조사 질문 항목**

**Survey Questionnaire**

**1. 본 연구에서는 통계적인 분석을 위해 연령, 성별, 진단받은 암 질환 정보 및 설문지 매칭, 답례품 증정을 위한 휴대폰 번호를 수집하되, 특정 개인을 추적하여 분석을 진행하지 않습니다.** **답례품은 설문에 참여해 주신 선착순 220명에게 제공되며, 답례품 발송 후 핸드폰 번호는 즉시 폐기되어 수집되지 않습니다. 연구대상자 설명문을 읽고 이해한 경우, 본 연구 참여에 관한 동의 여부를 아래에서 선택해 주십시오.**

**Age, gender, and diagnosed cancer information are collected in this study for statistical analysis. Mobile phone number will also be collected for questionnaire matching and gift presentation; however, the analysis will not be conducted by tracking specific individuals. A gift will be provided to 220 participants on a first-come, first-served basis, and mobile phone numbers will be immediately discarded upon gift presentation. If you have read and understood the instruction for the study subject, please select below whether or not you agree to participate in this study.**

① 동의 ② 비동의

**① Agree ② Not agree**

**2. 귀하가 소속된 암 환우 카페를 선택해 주십시오. 두 곳에 모두 가입하신 분은 둘 중 하나만 선택해 주시고, 설문 참여도 1회만 인정됩니다.**

**Please choose the cancer patient community you belong to. If you have joined both communities, please select either one. Only one survey participation will be accepted.**

① 암(만성병) 극복의 길을 찾는 사람들

② 구충제 후기 공유

**① People looking for a way to overcome cancer (chronic disease)**

**② Sharing my review of** taking anthelmintics

**3. 암 환우 카페 회원 닉네임 Community nickname**

( )

**4. 네이버 ID Naver ID**

( )

**5. 성별 Gender**

① 남성 ② 여성

**① Male ② Female**

**6. 귀하의 나이를 기재해 주십시오. Please write your age.**

( 세 ) ( years old)

**7. 귀하의 학력을 선택해 주십시오.**

**Please choose your educational background.**

① 무학

② 초졸

③ 중졸

④ 고졸

⑤ 대졸 이상

**① No formal education**

**② Elementary school graduate**

**③ Middle school graduate**

**④ High School graduate**

**⑤ College graduate**

**8. 진단 받은 암질환을 선택해 주십시오.**

**Please choose the cancer type you have been diagnosed with.**

① 폐암

② 위암

③ 간암

④ 대장암

⑤ 유방암

⑥ 기타 ( )

**① Lung cancer**

**② Stomach cancer**

**③ Liver cancer**

**④ Colorectal cancer**

**⑤ Breast cancer**

**⑥ Other ( )**

**9. 진단 받은 암의 병기를 선택해 주십시오.**

**Please choose the stage of the cancer at which you were diagnosed.**

① 1기

② 2기

③ 3기

④ 4기

**① Stage 1**

**② Stage 2**

**③ Stage 3**

**④ Stage 4**

**10. 암을 진단받은 시기는 언제였는지 연도와 월을 대략적으로 답변해 주시기 바랍니다.**

**Please mention the approximate year and month of your diagnosis with cancer.**

( 년 월)

(MM, YYYY)

**11. 항암목적으로 구충제 복용을 시작했던 시기는 언제였는지 연도와 월을 대략적으로 답변해 주시기 바랍니다.**

**Please mention the approximate year and month when you began taking anthelmintics for cancer treatment.**

( 년 월 )

(MM, YYYY)

**12. 항암목적으로 구충제를 얼마 동안 복용했는지 복용 기간을 대략적인 개월 수로 산정하여 답변해 주시기 바랍니다.**

**Please mention the approximate duration of treatment with anthelmintics for cancer treatment so far.**

약 ( ) 개월

about ( ) months

**13. 항암목적으로 구충제를 복용했던 시기에 대해 다음 중에서 선택해 주십시오. (복수답변 가능)**

**Please select the period(s) during which you have taken anthelmintics for cancer treatment (Multiple answers allowed).**

① 암을 진단받고 항암 약물치료를 시작하기 이전

② 암 치료를 위해 항암 약물치료와 병행하여

③ 암 치료를 마치고 항암 약물치료의 휴약(休藥) 상태일 때

④ 더 이상의 항암 약물치료가 불가하여 치료를 중단한 상태일 때

**① After diagnosis, before chemotherapy**

**② During chemotherapy**

**③ Resting chemotherapy**

**④ Discontinuing chemotherapy**

**14. 구충제를 암 치료 목적으로 현재도 복용하고 계시는지요?**

**Are you still taking anthelmintics for cancer treatment?**

① 예 ② 아니오

**① Yes ② No**

**15. 항암목적으로 구충제를 복용하게 된 동기에 대해 답변해 주시기 바랍니다.**

**Please mention what motivated you to take anthelmintics for cancer treatment.**

① 뉴스를 통해 소식 듣고

② 유튜브를 통해 소식 듣고

③ 인터넷 기사를 통해 소식 듣고

④ 암환자 환우회 카페를 통해 소식 듣고

⑤ 지인 소개를 통해

⑥ 의사의 권유

⑦ 기타 ( )

**① By hearing to information from TV news**

**② By hearing to information from YouTube**

**③ By obtaining information from online news**

**④ By obtaining information from online communities**

**⑤ By obtaining information from acquaintances**

**⑥ By recommendation of a clinician**

**⑦ Others ( )**

**16. 항암목적으로 복용했던 구충제의 구매처는 어디인지요? (여러 방법으로 구매했을 경우 복수답변 가능)**

**From where did you purchase anthelmintics for cancer treatment?**

**(Multiple answers allowed.)**

① 지역 약국

② 인터넷 구매

③ 기타 ( )

**① Local pharmacy**

**② Purchased online**

**③ Others ( )**

**17. 항암목적으로 복용했던 구충제 이름을 적어주시기 바랍니다. 모르실 경우 모른다고 써 주십시오.**

**Please mention the name of the anthelmintic you used for cancer treatment. If you do not know the name of the drug, please write ‘Do not know’.**

( )

**18. 항암목적으로 복용했던 구충제의 복용법을 선택해 주시기 바랍니다. (여러 방법으로 복용했을 경우 복수답변 가능)**

**Please choose the medication method of anthelmintics you have taken for cancer treatment (Multiple answers allowed).**

① 휴약(休藥) 없이 날마다 복용함

② 며칠 복용 후 며칠 휴약(休藥)하는 일정 주기에 따라 복용함

③ 간헐적으로 일정 주기 없이 복용함

④ 기타 ( )

**① Taken daily without resting**

**② In a routine schedule with resting**

**③ Taken it intermittently without a routine schedule**

**④ Others** ( )

**19. 항암목적으로 복용했던 구충제의 1일 복용법을 선택해 주시기 바랍니다. (여러 방법으로 복용했을 경우 복수답변 가능)**

**Please choose the daily dosing regimen of the anthelmintics you have taken for cancer treatment (If you have taken in various ways, multiple answers allowed).**

① 하루 1회

② 하루 2회

③ 하루 3회 이상

④ 모르겠다

**① Once a day**

**② Twice a day**

**③ More than three times**

**④ Do not know**

**20. 항암목적으로 복용한 구충제가 암 치료에 효과가 있었다고 생각하십니까?**

**Do you think the anthelmintics you have taken were effective in treating cancer?**

**(예 --> 21번 질문으로, 아니오 --> 22번 질문으로)**

**(Yes--> Go to question no. 21, No---> Go to question no. 22)**

① 예 ② 아니오

**① Yes ② No**

**21. 항암목적으로 복용한 구충제가 암 치료에 효과가 있었다면 그 이유는 무엇입니까? (복수답변 가능)**

**If the anthelmintics taken were effective in cancer treatment, why do you think so (Multiple answers allowed)?**

① 병원 검진결과 암 크기가 감소함

② 병원 검진결과 암 개수가 감소함

③ 스스로 느끼는 몸의 컨디션이 좋아짐

④ 기타 Others ( )

**① Decline in tumor size**

**② Decrease in the number of tumor masses**

**③ Improvement in physical condition**

**④ Others ( )**

**22. 항암목적으로 복용한 구충제가 효과가 없었다면 그 이유는 무엇입니까? (복수답변 가능)**

**If the anthelmintics taken were ineffective in cancer treatment, why do you think so (Multiple answers allowed)?**

① 병원 검진결과 암 상태에 변동 없음

② 병원 검진결과 암이 악화됨

③ 기타 ( )

**① No change in cancer status**

**② Worsening of cancer status**

**③ Others (** )

**23. 암 치료 목적으로 구충제를 복용 후 경험했던 부작용이 있습니까?**

**Have you experienced any adverse effects after taking anthelmintics for cancer treatment?**

**(예 --> 24번 질문으로, 아니오 --> 30번 질문으로)**

**(Yes--> Go to question no. 24, No.---> Go to question no. 30)**

① 예 ② 아니오

**① Yes ② No**

**24. 암 치료 목적으로 구충제를 복용 후 항암 치료 약물 부작용과는 별개로, 구충제로 인해 경험했다고 생각되는 부작용에 대해 선택해 주시기 바랍니다. (복수답변 가능)**

**Apart from the adverse effects of chemotherapy, please choose an adverse effect you think was caused by anthelmintics use (Multiple answers allowed).**

① 구역, 구토, 설사 등 위장관 증상

② 간독성

③ 혈액학적 증상

④ 기타 ( )

**① Gastrointestinal symptoms such as nausea, vomiting, and diarrhea**

**② Hepatotoxicity**

**③ Hematological symptoms**

**④ Others** ( )

**25. 항암목적으로 구충제를 복용 후 현재까지 구충제로 인해 경험하신 부작용 횟수를 선택해 주십시오.**

**Please choose the number of times that you have experienced adverse effects so far after taking anthelmintics for cancer treatment.**

① 1회

② 2회

③ 3회 이상

**① Once**

**② Twice**

**③ More** than three times

**26. 항암목적으로 구충제를 복용 후, 구충제로 인해 경험하신 부작용이 발생했던 시기를 선택해 주십시오. (부작용을 여러 번 경험했을 경우 복수답변 가능)**

**Please mention when the adverse effects occurred after taking anthelmintics for cancer treatment (If you have experienced several times, multiple answers allowed).**

① 첫 복용 이후 하루 이내에

② 첫 복용 이후 일주일 이내에

③ 첫 복용 이후 한 달 이내에

④ 첫 복용 이후 한 달 이상 지나서

**① within a day since the first use of anthelmintics**

**② within a week day since the first use of anthelmintics**

**③ within a month since the first use of anthelmintics**

**④ after more than one month has passed since the first use of anthelmintics**

**27. 항암목적으로 구충제를 복용 후, 구충제로 인해 나타났던 부작용이 지속되었던 기간을 선택해 주십시오. (부작용을 여러 번 경험했을 경우 복수답변 가능)**

**Please choose the duration of the adverse effects that you experienced after taking anthelmintics for cancer treatment (If you have experienced several times, multiple answers allowed).**

① 하루 정도 지속함

② 일주일 정도 지속함

③ 한 달 정도 지속함

④ 한 달 이상 지속함

**① persisted for a day**

**② persisted for about a week**

**③ persisted for about a month**

**④ persisted for more than a month**

**28. 항암목적으로 구충제를 복용 후 나타났던 부작용의 정도에 대해 선택해 주십시오. (부작용을 여러 번 경험했을 경우 복수답변 가능)**

**Please choose the severity of adverse effects you experienced after taking anthelmintics for cancer treatment (If you have experienced several times, multiple answers allowed).**

① 불편감은 느끼지 못했으나 혈액 검사 수치가 나빠짐

② 약간 불편한 정도

③ 불편하지만 참을 수 있는 정도

④ 부작용이 심각하여 일상생활에 어려움을 느끼는 정도

**① Not uncomfortable, but worsening of hematological parameters**

**② A bit uncomfortable**

**③ Uncomfortable, but endurable**

**④ Very severe, interfering with daily life**

**29. 항암목적으로 구충제를 복용 후, 구충제로 인한 부작용이 나타났을 때 취한 조치를 선택해 주십시오. (부작용을 여러 번 경험했을 경우 복수답변 가능)**

**Please select the action you took when you developed adverse effects after taking anthelmintic drugs for cancer treatment (Multiple answers allowed).**

① 구충제 복용을 중단함

② 구충제 종류를 변경함

③ 부작용이 있더라도 복용하던 구충제를 계속 복용함

④ 부작용을 완화하기 위해 다른 약물을 복용함

⑤ 기타 ( )

**① Discontinued anthelmintics**

**② Changed the type of anthelmintics**

**③ Continued the same anthelmintics**

**④ Used different additional medicines**

**⑤ Others ( )**

**30. 항암목적으로 구충제를 복용하고 있는 것에 대해 담당 의사도 알고 있었는지요?**

**Have you informed your clinician about taking anthelmintics to treat your cancer?**

**(예 --> 31번 질문으로, 아니오 --> 32번 질문으로)**

**(Yes--> Go to question no. 31, No.---> Go to question no. 32)**

① 예 ② 아니오

**① Yes ② No**

**31. 항암목적으로 구충제를 복용하는 것과 관련하여 담당 의사로부터 도움을 받은 사항이 있으시면 기재하여 주십시오. (복수답변 가능)**

**If you received any support from your clinician regarding the use of anthelmintics for cancer treatment, please choose below (Multiple answers allowed).**

① 복용하면 도움이 될 구충제 종류에 대한 조언

② 구충제 복용방법 지도

③ 구충제 복용 이후 부작용 발생에 따른 조치

④ 없음

⑤ 기타( )

**① Advice on choosing the anthelmintic type**

**② Guidance for anthelmintic medication**

**③ Actions to deal with adverse effects of anthelmintics**

**④ No support**

**⑤ Others ( )**

**32. 구충제 복용 경험과 관련하여 혹시 하시고 싶은 말씀이 있으시면 기재해 주십시오.**

**If you have any other points to mention regarding your experience taking anthelmintics, please write them here.**

( )

**33. 기프티콘 발송을 위해 귀하의 핸드폰 번호를 기재해 주십시오.**

**Please provide your mobile phone number for us to send the gift card.**

( )

설문에 참여해 주셔서 감사드립니다.

Thank you for participating in the survey.

- 본 연구는 구충제의 항암목적 사용을 권장하지 않으며 조사결과는 연구를 위한 자료로 활용됩니다. -

- This study does not recommend the use of anthelmintics for cancer treatment, and the results of the study will be used only for study purposes -
